# Rift Valley Fever in Namibia, 2010

**DOI:** 10.3201/eid1912.130593

**Published:** 2013-12

**Authors:** Federica Monaco, Chiara Pinoni, Gian Mario Cosseddu, Siegfried Khaiseb, Paolo Calistri, Umberto Molini, Alec Bishi, Annamaria Conte, Massimo Scacchia, Rossella Lelli

**Affiliations:** Istituto Zooprofilattico dell’Abruzzo e del Molise G. Caporale, Teramo, Italy (F. Monaco, C. Pinoni, G.M. Cosseddu, P. Calistri, U. Molini, A. Conte, M. Scacchia, R. Lelli);; Central Veterinary Laboratory, Windhoek, Namibia (S. Khaiseb);; Ministry of Agriculture Water and Forestry, Windhoek (A. Bishi)

**Keywords:** Rift Valley fever, Rift Valley fever virus, viruses, outbreaks, zoonoses, Namibia, South Africa

## Abstract

During May–July 2010 in Namibia, outbreaks of Rift Valley fever were reported to the National Veterinary Service. Analysis of animal specimens confirmed virus circulation on 7 farms. Molecular characterization showed that all outbreaks were caused by a strain of Rift Valley fever virus closely related to virus strains responsible for outbreaks in South Africa during 2009–2010.

Rift Valley fever virus (RVFV; family *Bunyaviridae*, genus *Phlebovirus*) is an enveloped RNA virus transmitted mainly by mosquitoes. This virus causes severe disease in humans and animals. The virus was identified in 1930 along the shores of Lake Naivasha in the Great Rift Valley in Kenya (*1**,**2*). Although direct transmission through contact with infected tissue might occur and could play a major role in human infection (*3*), mosquitoes still represent the most common way the virus is spread. Mosquito of several species (mainly *Culex* and *Aedes* spp.) have been considered vectors and reservoirs of the virus (*4**–**6*).

In 2010, South African veterinary authorities reported to the World Organisation for Animal Health 489 Rift Valley fever (RVF) outbreaks during the epidemic season; >14,000 cases and 8,000 deaths of animals occurred (*7**,**8*). The epidemic started on January 2010 in the eastern Free State Province and progressively spread west to Western Cape and Northern Cape Provinces and reached the border with Namibia. In Namibia, although virus circulation has been demonstrated in humans (*9**–**11*), little information is available on the distribution and the molecular characterization of RVFV circulating there. We conducted a study to identify and characterize RVFV strains that caused disease outbreaks in Namibia in 2010.

## The Study

During May 9–July 30, 2010, ovine and caprine flocks showing clinical signs compatible with RVFV infection were reported to Namibian Veterinary Service. Blood samples were collected from live animals, and liver, spleen, heart, uterus, kidney, and brain samples were obtained from dead animals. Samples were sent to the Central Veterinary Laboratory in Windhoek, Namibia, for laboratory analysis. Tissue samples (100 mg) were homogenized by using a mortar and sterile quartz pestle and diluted 1:10 in phosphate-buffered saline containing antimicrobial drugs (100 U/mL penicillin, 100 μg/mL streptomycin, 5 μg/mL gentamicin, 50 U/mL nystatin). Tissue debris was removed by low-speed centrifugation.

RNA was purified from blood samples and supernatants of homogenized tissues by using the High Pure Viral Nucleic Acid Extraction Kit (Roche Diagnostics, Mannheim, Germany) according to the manufacturer’s instructions. RVFV RNA was identified in samples by using the specific one-step reverse transcription PCR (RT-PCR) described by Battles and Dalrymple (*12*), which is specific for 369-nt region of the medium (M) segment of RVFV RNA. Laboratory tests confirmed circulation of RVFV on 7 farms in the Hardap and Karas regions (Figure).

**Figure:**
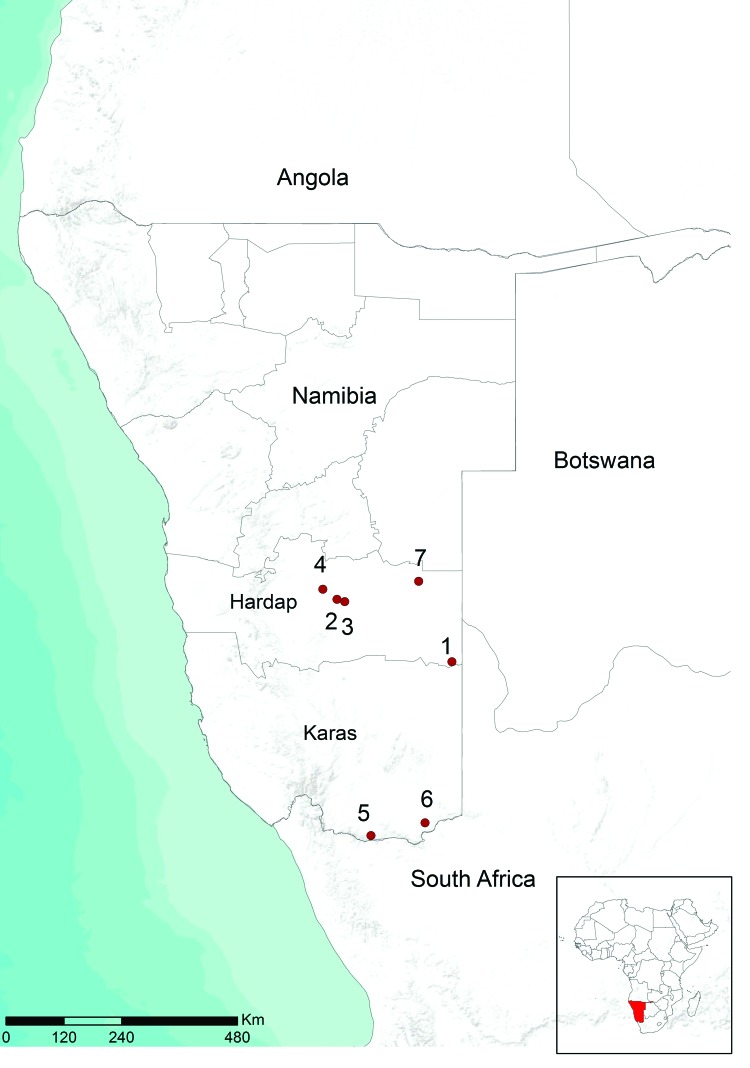
Location of farms in Namibia with Rift Valley fever virus infection, 2010. Red circles and numbers indicate outbreaks from which virus circulation was determined.

Aliquots of samples were shipped to the Istituto Zooprofilattico Sperimentale dell’Abruzzo e del Molise in Teramo, Italy, where virus isolation was conducted on samples positive for virus by RT-PCR by infecting Vero E6 cell (ATCC CRL-1586 VERO C1008) monolayers (Technical Appendix Table 1). RT-PCR amplicons from virus-positive samples were purified by using the QIAquick PCR Purification Kit (QIAGEN, Valencia, CA, USA) and used for direct sequencing. Sequencing was performed by using the Big Dye Terminator Kit (Applied Biosystems, Foster City, CA, USA). Excess dye was removed by using Cleanseq (Beckman Coulter, Inc., Brea, CA, USA). Nucleotide sequences were determined by using DNA sequencer ABI PRISM 3100 (Applied Biosystems). Amplification and sequencing were repeated twice to avoid introduction of artificial substitutions. Raw sequence data were assembled by using Contig Express (Vector NTI suite 9.1; Invitrogen, Carlsbad, CA, USA), and a 328-nt fragment of the Gn glycoprotein coding sequence were obtained after deletion of primer sequences.

Seven sequences were obtained, 1 from each of the 7 outbreaks. Sequences showed 100% similarity at nucleotide and amino acid levels. The entire sequence of the M segment of 2 isolates collected (1 in Hardap and 1 in Karas) (Technical Appendix Table 1) was generated after amplification of 9 overlapping sections. RT-PCR primers used are shown in Technical Appendix Table 2. Because sequences were 100% identical, the RVFV isolate (Namibia 2010), was considered representative of all isolates. The Basic Local Alignment Search Tool (www.ncbi.nlm.nih.gov) was used to identify homologous regions in sequence databases. Sequences were aligned by using ClustalW (www.clustal.org/) and BioEdit Sequence Analysis Editor version 7.0.5.3 (*13*). Phylogenetic analysis was conducted by using the entire sequence of the M segment from Namibia 2010 and all homologous sequences available in GenBank (Technical Appendix, Figure 1). Because of absence of entire sequences from strains that co-circulated in South Africa and Namibia in 2009–2010, we performed phylogenetic analysis of a 490-nt fragment by using a selection of reference strains that had been isolated in different years or countries (Technical Appendix Table 3) using the maximum-likelihood method in MEGA version 5 (*14*) with bootstrap support (1,000 replicates) (Technical Appendix Figure 2). The unique sequence generated was submitted to GenBank under accession no. KC935380.

Overall diversity of partial M segment sequences was low, and bootstrap values for tree nodes were weak in some instances. Phylogenetic analysis showed that isolate Namibia 2010 belongs to the same group of RVFV strains isolated in South Africa in 2009 (SA404/09) and 2010 (SA85/10, SA1224/10, SA373/10, SA1221/10, SA276/10, SA276/10, SA106/10, SA404/09, SA423/10, SA482/10, SA71/10, and SA54/10). The cluster corresponds to lineage H of RVFV identified by Grobbelaar et al. (*11*). SPU77/04, which was isolated from a human in Namibia in 2004, is closely related. The number of nucleotide differences between sequences of this group was low (0–3 nt), Isolate Namibia 2010 showed 100% nt identity with SA54/10 and a 1-nt difference with SA85/10, SA482/10, SA71/10, SA106/10, SA404/09, and SA423/10.

## Conclusions

The high degree of sequence identity of related RVFV strains that co-circulated in South Africa and Namibia in 2004–2010 suggests that these strains probably originated from a virus population that circulated between these 2 countries. Molecular data suggest that RFV outbreaks in Namibia in 2010 were caused by possible disseminated infections from South Africa. This hypothesis is further supported by the temporal and geographic location of the outbreaks. Clinical signs were first observed at the beginning of May in southeastern Hardap near the border with South Africa (Figure). The Auob River runs through this area, crosses the border with South Africa, and enters Kalahari National Park. Four outbreaks occurred in central Hardap (Figure) during the second half of May and the beginning of June in an area near the Auros River and an artificial lake in Hardap that supplies a broad system of water (irrigation) channels. During June 3–14, additional spread of virus was observed in the southern part of Karas near the border with South Africa where 2 outbreaks were confirmed (Figure), again near a water source, the Oranje River, which is the border between Namibia and South Africa.

The large RVF epidemic in South Africa in 2010 was attributed to heavy rainfall during January–February 2010 (*15*). In Namibia, evidence of intense rainfall was not recorded in the regions where disease outbreaks occurred in 2010 (Technical Appendix Figure 3). This finding indicates that reactivation of local virus circulation is unlikely. Our findings suggest that control measures along borders of Namibia and other countries should be reinforced and that collaborations between veterinary and public health authorities should be strengthened to reduce the effects of future outbreaks.

Technical Appendix Supplementary data for analysis of Rift Valley fever in Namibia, 2010.
