# Supplementary material for: Rift Valley Fever in Namibia, 2010
Source: Emerg Infect Dis. 2013 Dec;19(12):2025–7. doi: 10.3201/eid1912.130593 (PMC3840870; doi:10.3201/eid1912.130593)
Supplement: Technical Appendix — Supplementary data for analysis of Rift Valley fever in Namibia, 2010. [file 13-0593-Techapp-s1.pdf]

Article DOI: http://dx.doi.org/10.3201/eid1912.130593

Rift Valley Fever in Namibia, 2010

Technical Appendix

Supplementary Data for Analysis of Rift Valley Fever in Namibia, 2010

Technical Appendix Table 1. Rift Valley fever outbreaks, Namibia, 2010*

				Diagnostic results		Medium segment sequence	
Outbreak	Region	Date of report	Animal	RT-PCR†	Virus isolation	Partial 328 nt	Full-length 3,885 nt
1	Hardap	2010 May 5	Sheep	+	+	+	–
2	Hardap	2010 May 15	Sheep	+	+	+	+
3	Hardap	2010 May 15	Sheep	+	+	+	–
4	Hardap	2010 May 22	Goats	+	–	+	–
5	Karas	2010 Jun 3	Sheep	+	+	+	+
6	Karas	2010 Jun 9	Sheep	+	+	+	–
7	Hardap	2010 Jun 14	Sheep	+	+	+	–

*RT-PCR, reverse transcription PCR.
†Methods for blood and tissues reported by Battles and Dalrymple (*1*).

Technical Appendix Table 2. Primers used for amplification and sequencing of the entire medium RNA segment of Namibia_10 RFV isolate from Namibia, 2010

RVFV section	Primer	Sequence, 5′→3′
1	RVFM-AFwd	ACACAAAGACGGTGC
	RVF_M_518R	TGCCCCTTCCCTGGTCTGT
2	RVF_M_427F	TGACAGTCCTTCCAGCCTTAGCAG
	RVF_M_990R	CTTCGCAGACCCCTTTCATTTTTG
3	RVF_M_821F	TTCAGTCAAGTGCCCTCCTAAG
	RVF_M_1356R	GTATCTGCACAATCCCTGACC
4	RVF_M_1262F	TGGGGACGCAGCATTTTG
	RVF_M_1713R	GCACTAAGCACGGGTCCTG
5	RVF_M_1629F	ATAGGGGTTCACATGGCACACGA
	RVF_M_2231R	GACCCCCTTCAACATCAAACAA
6	RVF_M_2105F	TCAGGCAAGCTCCAGAATC
	RVF_M_2702R	TGCGTCCAGTGAGAGGCTAAC
7	RVF_M_2577F	ATCGACTGGGTGCATAAACTCA
	RVF_M_3107R	ACAAGATACGGCTGCTCCCACAAA
8	RVF_M_2866F	GGGCACCAAACCTTATCTCAT
	RVF_M_3601R	TTAGTAGCAGCAAGCCACATTTT
9	RVF_M_2866F	GGGCACCAAACCTTATCTCAT
	RVFM-ARev	ACACAAAGACCGGTGC

*RVFV, Rift Valley fever virus.

Technical Appendix Table 3. Reference strains used in phylogenetic analysis of Rift Valley fever virus, Namibia, 2010*

Isolate	Country and year of collection	GenBank accession no.
Namibia 2010	Namibia, 2010	KC935380
SPU204/85	Angola, 1985	HM587076
ARD-38388	Burkina Faso, 1983	DQ380187
ArB1976	CAR, 1969	HM587083
73HB1230	CAR, 1973	DQ380221
73HB1449	CAR, 1973	DQ380211
74HB59	CAR, 1974	HM587082
Hv-B375	CAR, 1985	DQ380218
CAR R1662	CAR, 1985	HM587086
CAR R1752	CAR, 1986	HM587087
AnK3837	Guinea, 1981	HM587084
ZH-548_	Egypt, 1977	AF134508
ZH-1776	Egypt, 1978	DQ380203
ZM-657	Egypt, 1978	DQ380204
ZS-6365	Egypt, 1979	DQ380205
93-Abeer	Egypt, 1993	HM587043

Isolate	Country and year of collection	GenBank accession no.
94EG Tambul	Egypt, 1994	HM587042
95EG Cow-2509	Egypt, 1995	HM587115
KEN57 Rintoul	Kenya, 1951	HM587104
B314	Kenya, 1962	HM587105
B674	Kenya, 1963	HM587106
S35	Kenya, 1972	HM587045
214445	Kenya, 1973	HM587074
Kitale1840	Kenya, 1974	HM587116
B309	Kenya, 1977	HM587070
B1143	Kenya, 1977	HM587075
SPU384/97/1	Kenya, 1997	HM587052
SPU2/98/9	Kenya, 1998	HM587055
Kenya 9800523	Kenya, 1998	DQ380196
SPU22/07/118	Kenya, 2007	HM587062
SPU22/07/125	Kenya, 2007	HM587063
SPU22/07/4	Kenya, 2007	HM587068
SPU22/07/129	Kenya, 2007	HM587064
MgH824	Madagascar, 1979	HM587040
MgAr811	Madagascar, 1979	HM587041
MgAn1002	Madagascar, 1991	HM587057
MgAn991	Madagascar, 1991	HM587060
M48/08	Madagascar, 2008	HQ009512
OS-1	Mauritania, 1987	DQ380186
OS-4	Mauritania, 1988	HM587122
H1 MAU 03	Mauritania, 2003	EF160116
H2 MAU 03	Mauritania, 2003	EF160115
SPU77/04	Namibia, 2004	HM587100
An278	Saudi Arabia, 2000	HM587050
Saudi 2000–10911	Saudi Arabia, 2000	DQ380197
HD21955	Senegal, 1975	HM587123
ArD38661	Senegal, 1983	HM587124
SPU12/99/21	Somalia, 1999	HM587051
SA51	South Africa, 1951	HM587125
Ar74	South Africa, 1955	HM587109
Ar118	South Africa, 1955	HM587120
An1830	South Africa, 1956	HM587108
Ar12568	South Africa, 1971	HM587112
35/74	South Africa, 1974	JF784387
H1739	South Africa, 1975	HM587110
H1825	South Africa, 1975	HM587114
SA-75	South Africa, 1975	DQ380189
Ar20364	South Africa, 1981	HM587101
SPU52/99/1	South Africa, 1999	HM587046
SPU52/99/2	South Africa, 1999	HM587048
SPU52/99/5	South Africa, 1999	HM587047
SPU52/99/3	South Africa, 1999	HM587049
SA52/08	South Africa, 2008	HM587069
M37/08	South Africa, 2008	HM587067
SPU86/09	South Africa, 2009	HM587065
SA404/09	South Africa, 2009	HM587096
SA85/10	South Africa, 2010	HM587098
SA1224/10	South Africa, 2010	HM587099
SA373/10	South Africa, 2010	HM587097
SA1221/10	South Africa, 2010	HM587090
SA276/10	South Africa, 2010	HM587093
SA276/10	South Africa, 2010	HM587093
SA106/10	South Africa, 2010	HM587094
SA482/10	South Africa, 2010	HM587089
SA54/10	South Africa, 2010	HM587092
SA71/10	South Africa, 2010	HM587088
SA184/10	South Africa, 2010	HM587107
SA423/10	South Africa, 2010	HM587095
SA59/10	South Africa, 2010	HM587091
95EG vaccine	Uganda, 1944	HM587103
Entebbe	Uganda, 1944	DQ380191
Lunyo	Uganda, 1955	HM587119
SPU44/85	Zambia, 1985	HM587079
Ar11186	Zimbabwe, 1969	HM587113
VRL763/70	Zimbabwe, 1970	HM587111
2250/74	Zimbabwe, 1974	DQ380209
2269/74	Zimbabwe, 1974	DQ380222
VRL2373/74	Zimbabwe, 1974	HM587121
VRL2051/76	Zimbabwe, 1976	HM587072
VRL1290/78	Zimbabwe, 1978	HM587077
VRL1548/78	Zimbabwe, 1978	HM587059

Isolate	Country and year of collection	GenBank accession no.
VRL2354/78	Zimbabwe, 1978	HM587058
VRL1217/78	Zimbabwe, 1978	HM587073
VRL1032/78	Zimbabwe, 1978	HM587085
VRL1290A/78	Zimbabwe, 1978	HM587078
VRL1887/78	Zimbabwe, 1978	HM587080
VRL1260/78	Zimbabwe, 1978	HM587081
VRL1516/78	Zimbabwe, 1978	HM587044
VRL1187/79	Zimbabwe, 1979	HM587056
VRL2413/98	Zimbabwe, 1998	HM587054

*CAR, Central African Republic.

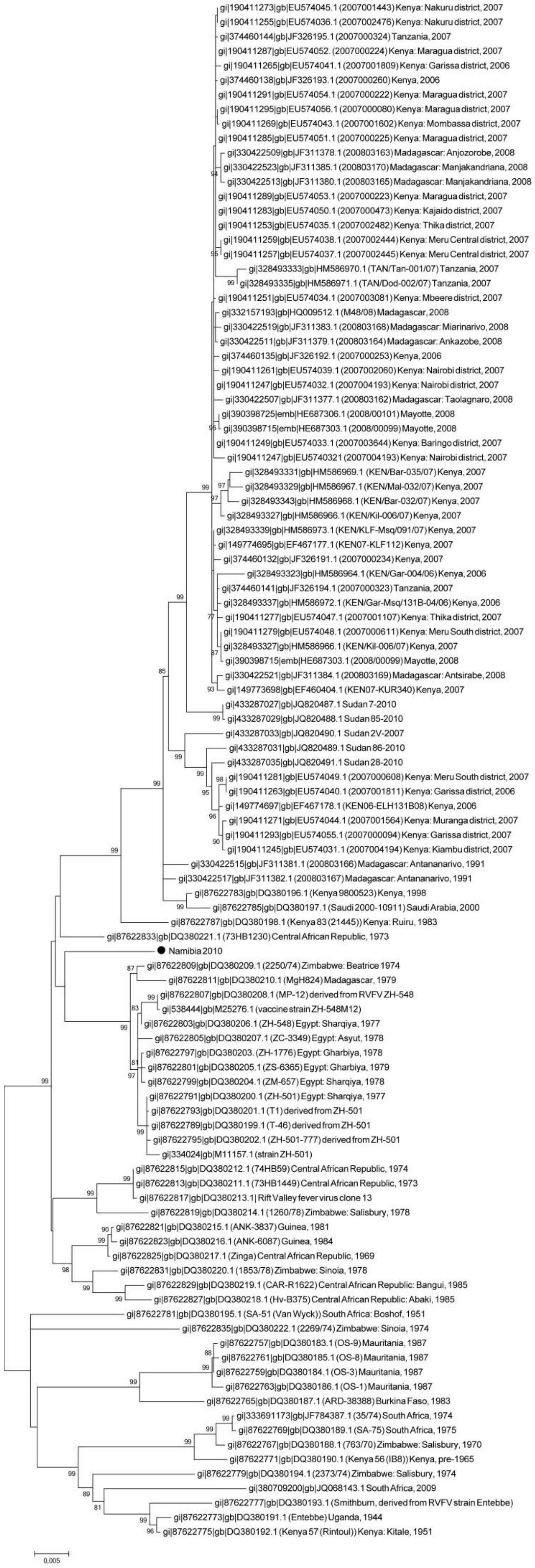

Technical Appendix Figure 1. Phylogenetic tree of complete sequences of Rift Valley fever virus medium RNA segment. Black circle indicates strain isolated in this study. Analysis was performed by using MEGA 5 software (*2*) and the maximum-likelihood method. Bootstrap support values >70 are shown along the branches (1,000 replicates). Scale bar indicates nucleotide substitutions per site.

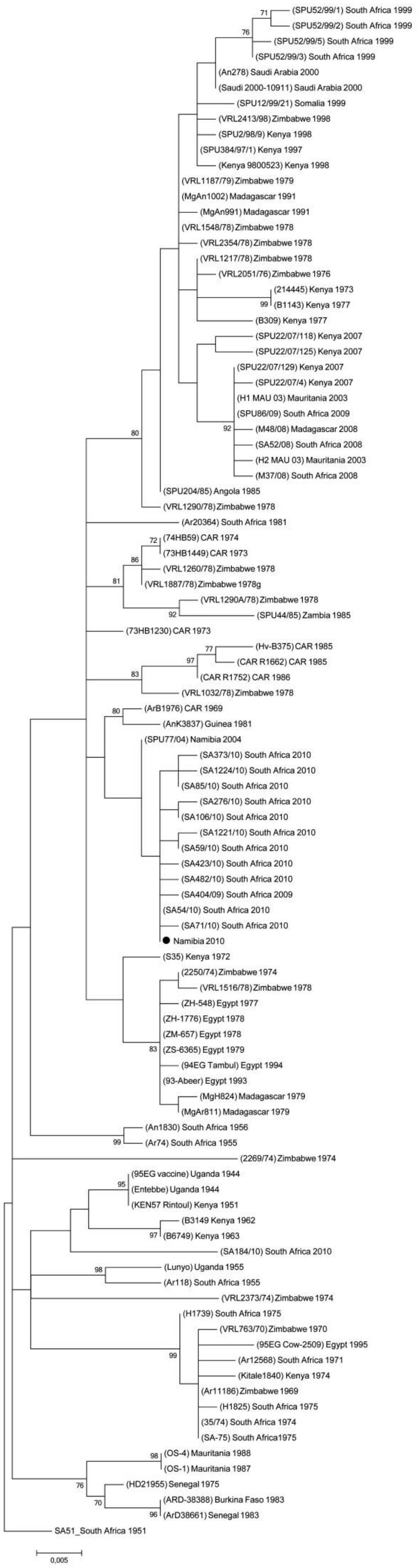

Technical Appendix Figure 2. Phylogenetic tree showing genetic relationships among Rift Valley fever virus (RVFV) isolates. The tree was constructed on the basis of 490-nt sequences of RVFV medium segment. The unique sequence obtained in this study is indicated with a black spot. Viruses are identified by country, year of collection, and nomenclature of RVFV isolates. Black circle indicates strain isolated in this study. GenBank accession numbers are shown in the Technical Appendix. Analysis was performed by using MEGA 5 software (*2*) and the maximum-likelihood method. Bootstrap support values >70 are shown (1,000 replicates) along the branches. Scale bar indicates nucleotide substitutions per site.

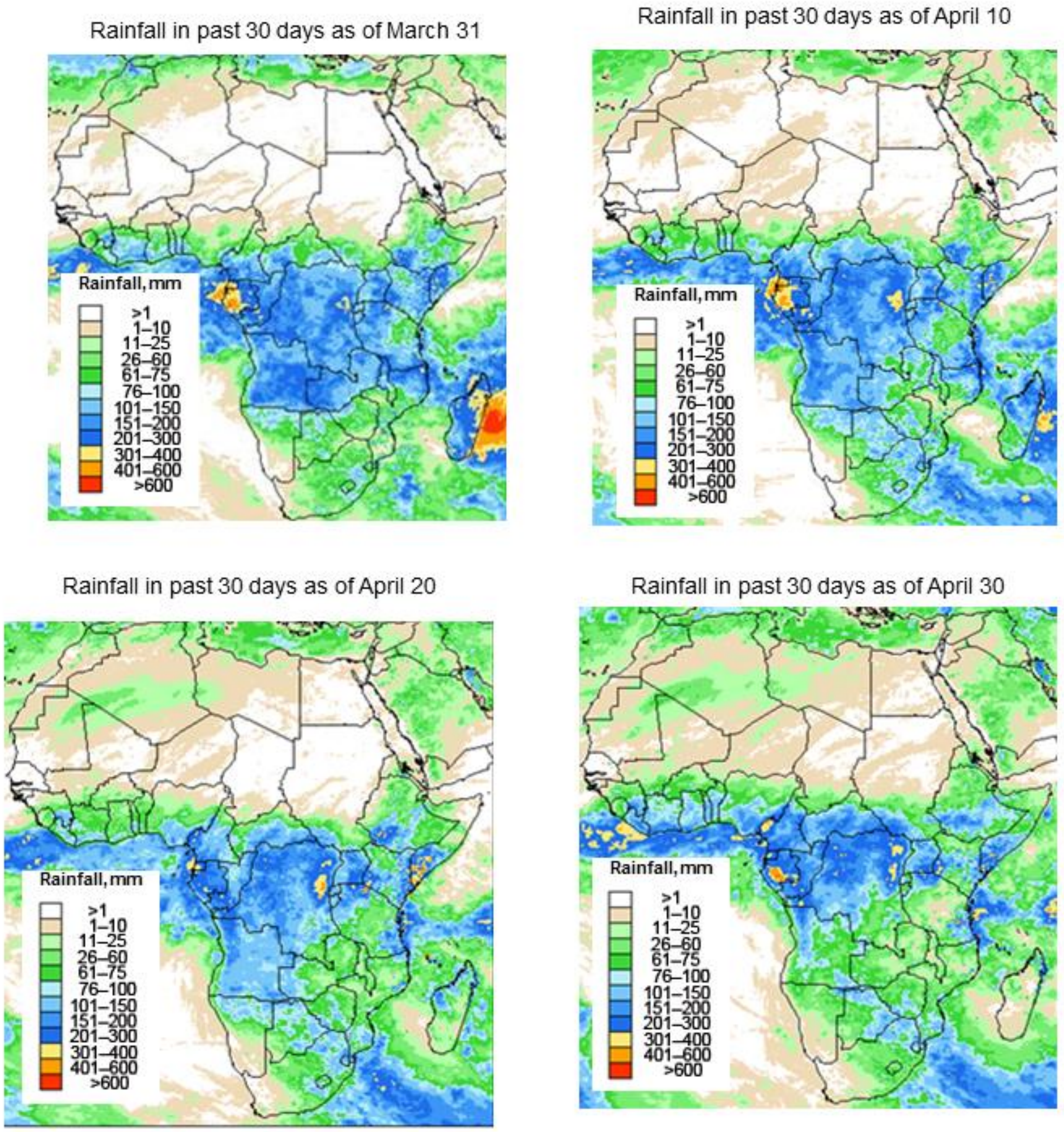

Technical Appendix Figure 3. Rainfall in Africa during the period before outbreaks of Rift Valley fever in Namibia started in May 2010.

References

1. Battles JK, Dalrymple JM. Genetic variation among geographic isolates of Rift Valley fever virus. Am J Trop Med Hyg. 1988;39:617–31. PubMed

2. Tamura K, Peterson D, Peterson N, Stecher G, Nei M, Kumar S. MEGA5: molecular evolutionary genetics analysis using maximum likelihood, evolutionary distance, and maximum parsimony methods. Mol Biol Evol. 2011;28:2731–9. PubMed http://dx.doi.org/10.1093/molbev/msr121
